# SEM Analysis of Surface Impact on Biofilm Antibiotic Treatment

**DOI:** 10.1155/2017/2960194

**Published:** 2017-01-11

**Authors:** Luciana Calheiros Gomes, Filipe José Mergulhão

**Affiliations:** LEPABE, Department of Chemical Engineering, Faculty of Engineering, University of Porto, Porto, Portugal

## Abstract

The aim of this work was to use scanning electron microscopy (SEM) to investigate the effect of ampicillin treatment on* Escherichia coli* biofilms formed on two surface materials with different properties, silicone (SIL) and glass (GLA). Epifluorescence microscopy (EM) was initially used to assess biofilm formation and killing efficiency on both surfaces. This technique showed that higher bacterial colonization was obtained in the hydrophobic SIL than in the hydrophilic GLA. It has also shown that higher biofilm inactivation was attained for GLA after the antibiotic treatment (7-log reduction versus 1-log reduction for SIL). Due to its high resolution and magnification, SEM enabled a more detailed analysis of the antibiotic effect on biofilm cells, complementing the killing efficiency information obtained by EM. SEM micrographs revealed that ampicillin-treated cells have an elongated form when compared to untreated cells. Additionally, it has shown that different materials induced different levels of elongation on cells exposed to antibiotic. Biofilms formed on GLA showed a 37% higher elongation than those formed on SIL. Importantly, cell elongation was related to viability since ampicillin had a higher bactericidal effect on GLA-formed biofilms. These findings raise the possibility of using SEM for understanding the efficacy of antimicrobial treatments by observation of biofilm morphology.

## 1. Introduction

Bacteria and other microorganisms tend to attach to solid surfaces where they grow and produce extracellular polymeric substances (EPS), forming a biofilm [1, 2]. Biofilms constitute a serious problem for public health particularly due to their potential to cause infections in patients with indwelling medical devices (IMDs) [3, 4] and the increased resistance of biofilm-associated microorganisms to antimicrobial agents [5].

Bacterial colonization and subsequent biofilm formation on IMD are dynamic and complex processes that are strongly influenced by the properties of the adhesion surface. Certain materials used in the design of IMDs are more prone to bacterial adhesion/biofilm formation than others. Surface characteristics determining the adherence properties of specific materials include the surface free energy, charge, hydrophobicity, and roughness [6]. Bacterial cells, which tend to have hydrophobic cell surfaces, are typically attracted to the hydrophobic surfaces of many biomaterials currently used in IMDs, such as silicone [7]. Although silicone is relatively more prone to bacterial attachment, biofilm formation, nonspecific adhesion of proteins, and biomolecules than many other polymers, it is commonly used in urinary catheters, contact lenses, breast implants, endotracheal tubes, and voice prosthesis [8, 9].

Biofilm eradication from biomedical devices is difficult mainly due to their resistance to antimicrobial agents. Once infected, the IMDs are often removed and replaced, causing a significant increase in the health care cost and chance of reinfection [10]. Considerable research endeavor is currently directed towards producing anti-infective and antiadhesive devices or implants by either (a) modification of material surface features (plasma and brushes), (b) anti-infective agents bound to the surface of the material (silver, quaternary ammonium compounds, synthetic antibiotics, and biosurfactants), or (c) release of soluble toxic agents (chlorhexidine, antibiotics) into the IMD surroundings [11, 12].

Several medical devices have different parts which are made from different materials bound together by transition seals. These transition zones are particularly attractive for bacterial adhesion and biofilm development. This type of mixed-surface devices is found in dental [13], orthopedic [14], and cardiovascular implants [15]. For example, FDA approved the use of abdominal [16] and coronary stents [17] with portions of polymeric and metallic materials.

Much of the current knowledge about biofilms is due to advances in microscopic imaging techniques. Standard optical microscopy, epifluorescence microscopy (EM), and confocal laser scanning microscopy (CLSM) are the most commonly used techniques for biofilm analysis. Nevertheless, scanning electron microscopy (SEM) has been shown to be a suitable tool not only to observe in detail the substratum morphology, but also to follow the bacterial adhesion and biofilm formation on abiotic surfaces. Indeed, electron microscopy has been used from an early age for examination and characterization of biofilms on medical devices [18, 19] and currently it has been useful in the development of antibiofilm materials for biomedical applications [20–22]. SEM has the level of magnification and resolution necessary to enable the observation of the overall shape of microorganisms composing the biofilm, as well as their spatial organization [23, 24]. This type of spatial analysis provided by SEM makes it an interesting method to assess the biofilm growth on mixed surfaces (in which there is a junction between two materials), unlike the traditional methods that provide a bulk quantification. Although SEM is not compatible with the use of fluorochromes, such as Syto9 and propidium iodide, commonly used to distinguish between viable and nonviable cells in EM, it enables the detailed observation of individual cells in the biofilm and their morphology [25, 26], in opposition to EM that lacks the required magnification and resolution.

Biofilm control in medical settings is a difficult challenge, particularly in the case of IMDs where the colonized surface is not readily accessible [27, 28]. Success in this war against biofilms requires a deeper understanding on the interactions between biofilm cells, the surface, antibiotics, and the host [27]. This work shows that the use of SEM for high resolution imaging of colonized surfaces can provide valuable information about the effect of surface properties on antibiotic treatment performance. Understanding these effects may provide clues for the fine-tuning of surface properties in biomedical materials in order to increase the efficiency of antimicrobial therapy.

## 2. Materials and Methods

### 2.1. Bacterial Strain and Culture Conditions

*Escherichia coli* JM109(DE3) from Promega (USA) was used in this study because this strain has shown a good biofilm forming ability in both turbulent [29, 30] and laminar [31] flow conditions and in different biofilm platforms [30–32]. A bacterial suspension was prepared by inoculation of 500 *μ*L of a glycerol stock (kept at −80°C) in a total volume of 0.2 L of inoculation medium previously described by Teodósio et al. [29]. This consisted of 5.5 g/L glucose, 2.5 g/L peptone, and 1.25 g/L yeast extract in phosphate buffer (1.88 g/L KH_2_PO_4_ and 2.60 g/L Na_2_HPO_4_), pH 7.0. This culture was grown on a 1 L shake-flask, incubated overnight at 37°C with agitation. Subsequently, cells were harvested by centrifugation (at 3202*g* for 10 min) and suspended in Mueller-Hinton broth (Merck, Germany) to remove all traces of the overnight growth medium. Cells were again harvested by centrifugation and suspended in Mueller-Hinton broth in order to obtain an inoculum containing approximately 1 × 10^7^ cells/mL.

### 2.2. Surface Preparation and Characterization

Square coupons of 1 cm^2^ made from glass (GLA, Vidraria Lousada, Lda, Portugal) and silicone (SIL, Neves & Neves, Lda, Portugal) were prepared. They were washed with a solution of 5% (v/v) commercial detergent (Sonasol Pril, Henkel Ibérica S.A.) for 30 min and then rinsed in ultrapure water to remove any remaining detergent [33, 34]. After air-drying the surfaces for 1 h, they were immersed in 96% (v/v) ethanol for 30 min in the case of GLA and 10 s in the case of SIL [35]. Then, the GLA coupons were autoclaved for 15 min at 121°C [33], whereas SIL coupons were autoclaved for 20 min at 70°C [34].

The water contact angles of the surfaces (*θ*_*w*_) were automatically determined by the sessile drop method in a contact angle meter (OCA 15 Plus, Dataphysics, Germany). The measurements for each material were performed in three independent experiments with at least 25 determinations on each surface.

### 2.3. Biofilm Formation

Each well of sterile 12-well polystyrene (PS), flat-bottomed microtiter plates (Orange Scientific, USA) containing the coupons was filled with 2 mL of the cell suspension previously prepared (1 × 10^7^ cells/mL in Mueller-Hinton broth). To promote biofilm formation, the plates were incubated at 37°C without shaking for 24 h. The 24 hour-time point was chosen because a previous study showed that* E. coli* biofilms formed on urinary catheters made of silicone were completely developed in 24 h [36].

### 2.4. Biofilm Susceptibility

The antibiotic used in this study was ampicillin (AppliChem, Germany), which is a *β*-lactam antibiotic that functions by blocking a specific cross-linking step in the cell wall production. This cross-linking failure creates weak bacterial cell walls that cannot sustain the cytoplasm, inducing cell lysis [37].

Biofilms were exposed to 5 × biofilm MIC of ampicillin (250 *μ*g/mL) for 7.5 h and coupons of each material were analysed every 1.5 h. The nonadherent cells were washed from the surfaces by immersion in sterile saline (8.5 g/L NaCl). In order to resuspend and homogenize the sessile cells, the washed surfaces were vortexed in 10 mL of saline solution during 1 min [38]. The level of total cell removal from the coupons was assessed by direct staining of untreated biofilms with 4′,6-diamidino-2-phenylindole (DAPI) [39] before and after vortexing to quantify the remaining cells. This efficiency was found to be 95% for GLA and 94% for SIL coupons (see Supplementary Material available online at https://doi.org/10.1155/2017/2960194). For cell viability assessment, the suspended cells were stained with the Live/Dead® (L/D) BacLight™ bacterial viability kit (Syto9/propidium iodide, Invitrogen Life Technologies, Alfagene, Portugal) [40]. Bacterial observation was performed after 10 min incubation with the fluorescent dyes in the dark using a Leica DM LB2 epifluorescence microscope connected to a Leica DFC300 FX camera (Leica Microsystems Ltd., Switzerland). Viable and total (viable plus nonviable) cell numbers were estimated on each membrane from counts of a minimum of 20 fields of view and the viability results were expressed as the mean of triplicate samples obtained in three independent experiments measured as log viable cells/cm^2^.

### 2.5. SEM

The cell morphology of* E. coli* biofilms present on GLA and SIL coupons before and after 6 h of antibiotic treatment was assessed by SEM. Prior to observations, biofilm samples were fixed using 3% wt. glutaraldehyde in cacodylate buffer pH 7.2 [41] for 10 min and exposed to an ethanol dehydration series of 50, 60, 70, 80, 90, and 2 × 100% (v/v) ethanol, followed by a chemical dehydration series of 100% ethanol + hexamethyldisilazane (HMDS, Ted Pella, USA) at 50, 60, 70, 80, 90, and 2 × 100% (v/v) HMDS [42], for 5 min at each concentration. The bare surfaces were also subjected to the same dehydration treatment. All the coupons were then dried for 1 day and sputter-coated with a palladium-gold thin film [43]. The bare surfaces and the biofilm samples were viewed with a SEM/EDS system (FEI Quanta 400FEG ESEM/EDAX Genesis X4M, FEI Company, USA) in high-vacuum mode at 15 kV. In the case of biofilm samples, twenty images from three independent coupons were analysed before and after the antibiotic treatment. The microscope software (xT Microscope Control, FEI Company, USA) was used to determine the cell length by measuring 100 randomly selected cells in each condition.

### 2.6. Statistical Analysis

The susceptibility assay was compared using one-way analysis of variance (ANOVA) based on a confidence level of 99% (differences reported as significant for *P* values < 0.01). Paired *t*-test analysis was also performed when appropriate.

## 3. Results

The water contact angles for both surfaces were determined and the value obtained with GLA (*θ*_*w*_ = 47.0 ± 0.4°) was smaller than the one obtained with SIL (*θ*_*w*_ = 115.4 ± 0.4°).

**Figure 1:**
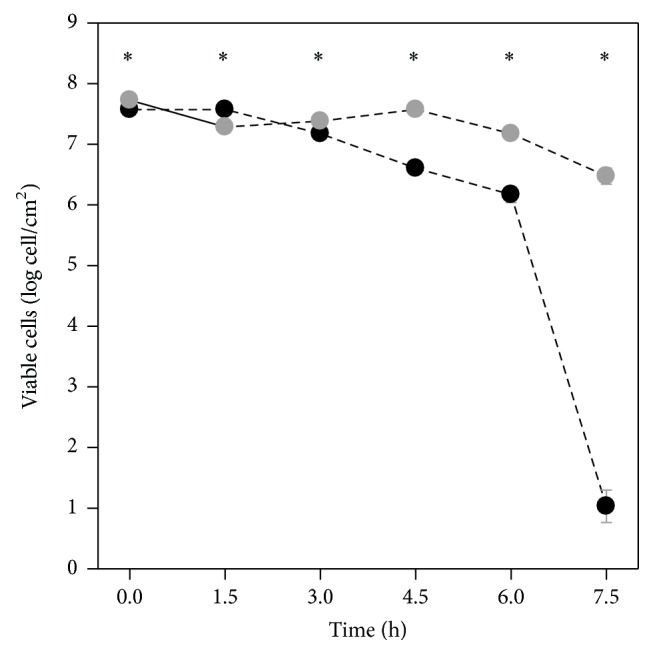
Evolution of the amount of viable cells within 24-hour biofilms formed on GLA (black circles) and SIL (grey circles) during exposure to ampicillin. Statistically significant differences between the materials (for a confidence level greater than 99%, *P* < 0.01) are pointed as *∗*. The means ± SDs for three independent experiments are illustrated.

Epifluorescence microscopy was used to quantify the biofilm formation and antibiotic susceptibility on both GLA and SIL surfaces. SIL surfaces had enhanced biofilm formation (16%) when compared to glass (7 × 10^7^ versus 6 × 10^7^ cells/cm^2^, *P* < 0.05) after 24 h of biofilm development.  Figure 1 presents the susceptibility curves of* E. coli* biofilms formed on both materials to a concentration equivalent to 5 × biofilm MIC of ampicillin. The number of viable cells remained constant for both materials during the first 3 h of experiment, but from this moment onwards, the viability of biofilms formed on GLA markedly decreased and a 7-log reduction was obtained after 7.5 h of treatment. Biofilms formed on SIL were much more resistant to ampicillin than those formed on GLA, with a reduction of only 1 log in the amount of viable bacteria (Figure 1).

**Figure 2:**
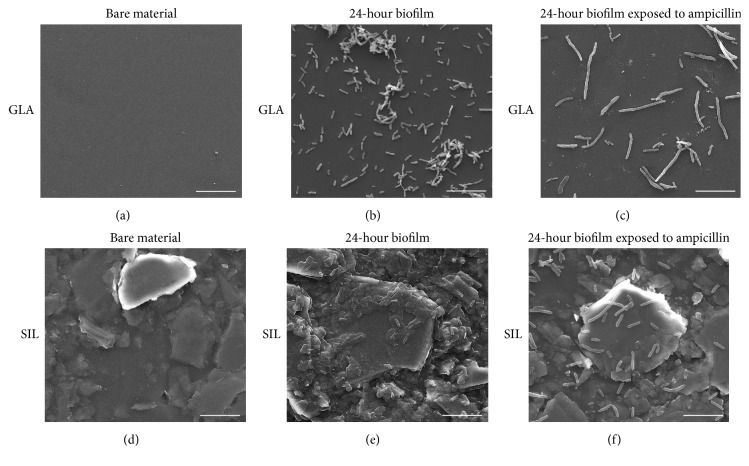
Scanning electron micrographs of bare surfaces ((a) GLA and (d) SIL) and 24-hour biofilms not exposed to ampicillin (formed on (b) GLA and (e) SIL) and after 6 h of exposure to ampicillin ((c) GLA and (f) SIL). GLA, glass; SIL, silicone. Magnification: 5000x; bars = 10 *μ*m.

SEM was the chosen method for analysing the bare surfaces and the morphological changes on the sessile cells exposed to ampicillin (Figure 2).

The micrographs of bare surfaces showed that GLA is a very smooth surface (Figure 2(a)), while SIL is a rough surface with a considerable number of microscale bumps and protrusions (Figure 2(d)). The size of these protrusions is quite variable (with features up to 20 *μ*m), although most of them exceed the size of* E. coli* cells adhered to the silicone surface (Figure 2(e)).

Regarding biofilm formation, SIL was the material showing the higher number of adhered cells (Figure 2(e)), confirming the results obtained by EM (not shown). On the other hand,* E. coli* cells adhered to SIL appear to be embedded in EPS (Figure 2(e)), in opposition to the cells observed on the GLA surface (Figure 2(b)) which are arranged in the form of aggregates or simply as individualized cells without slimy material in their vicinity. After 6 h of antibiotic exposure, the amount of biofilm cells adhered to both GLA and SIL coupons decreased (Figures 2(c) and 2(f), resp.). Additionally, SEM micrographs revealed that ampicillin-treated cells are more elongated on both materials when compared to untreated cells and that, in the case of biofilms developed on GLA (Figure 2(c)), the treated cells are longer than on SIL (Figure 2(f)). Interestingly, in both tested materials, the cell wall of sessile cells does not show signs of severe damage after 6 h of ampicillin treatment (Figures 2(c) and 2(f)).

**Figure 3:**
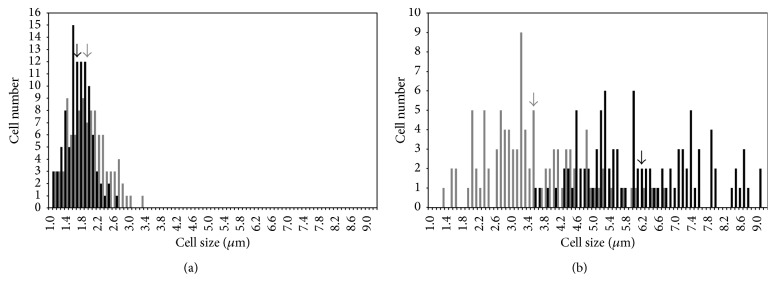
Cell length distribution of 24-hour biofilms not exposed to ampicillin (a) and after 6 h of exposure to ampicillin. GLA (black bars) and SIL (grey bars). The arrows represent the average cell length determined from SEM micrographs for each experimental condition.

Determination of the cell length from SEM micrographs resulted in histograms showing the size distribution of biofilms cells exposed (Figure 3(b)) and not exposed to ampicillin (Figure 3(a)). Whereas nonexposed cells adhered to both materials had similar lengths (approximately 1.8 *μ*m, Figure 3(a)), treated cells present on GLA coupons were more elongated (37%) than those present on SIL surfaces (Figure 3(b)). Additionally, after antibiotic treatment (Figure 3(b)), cells adhered to SIL measured between 1.3 and 6.7 *μ*m (on average 46% longer than the SIL-untreated cells), while cell lengths between 3.6 and to 9.1 *μ*m were determined for GLA (on average 73% longer than the GLA-untreated cells). Also a much narrower size distribution was found for the untreated cells, regardless of the material tested.

## 4. Discussion

The main goal of this study was to investigate the impact of different surface materials on* E. coli* biofilm formation and antibiotic treatment. Contact angle measurements have shown that GLA is a hydrophilic surface (*θ*_*w*_ < 65°), whereas SIL is a hydrophobic surface (*θ*_*w*_ > 65°) [44].

The results of EM and SEM indicated that a higher amount of biofilm was formed on the hydrophobic silicone surface. Many authors [45–48] have shown that silicone is highly prone to colonization by* E. coli*, although it is widely used in biomedical devices. Our findings suggest that the substratum hydrophobicity positively affected the bacterial adhesion, as shown in previous studies [35, 49, 50]. In addition to hydrophobicity, the irregularity of the SIL surface is another physical property that may have led to increased cellular adhesion. It has been reported that irregularities in polymeric surfaces promote bacterial adhesion and biofilm formation, unlike the smooth surfaces that do not favor bacterial deposition [51]. That probably happens because a rough surface has a greater surface area and the depressions in roughened surfaces provide more favorable sites for colonization [52, 53].

Regarding biofilm susceptibility, the EM analysis showed that biofilms developed on SIL were less susceptible to ampicillin than those formed on GLA. This may be due to their higher cell density (number of cells per unit area) and/or presence of EPS when compared to the biofilms formed on GLA. It has been shown that the spatial arrangement of a higher number of cells can create concentration gradients (of nutrients, antibiotic, and oxygen) within the biofilm structure [54], contributing to the decreased antibiotic susceptibility [55, 56]. Additionally, it has been described that the relative efficacy of some antimicrobial agents declines with the cell density [57–60]. Also it is known that EPS are extremely important for protecting biofilm cells from antibiotics [59, 61], and since in this work EPS were only observed on SIL coupons, they may have contributed to an increased biofilm resilience. Hence, it can be concluded that the surface properties influenced the killing efficiency of the antimicrobial agent against biofilms, as observed in previous studies [62–65]. Gristina et al. [62] demonstrated that the degree of bacterial colonization and antibiotic resistance are associated with the biomaterial and may be altered by biomaterial-induced phenotypic changes. Later, Webb et al. [63] found that the surface-adherent mode of bacterial growth determines the antibiotic resistance of biofilms. It was also reported that some materials are responsible for selecting variant adhesive bacteria with increased antibiotic resistance [64].

SEM was used in this study with the purpose of complementing the results obtained by EM, providing a more detailed analysis of the antibiotic effect on biofilms. Due to its high resolution and magnification, SEM revealed that biofilm cells exposed to ampicillin had an elongated morphology. It is well documented that antibiotics can affect bacteria in ways other than the expected bactericidal action, such as inducing morphological changes [48, 66–69]. A common response of Gram-negative bacteria to the effects of *β*-lactam antibiotics is an abnormal elongation of the individual cells [67]. This type of morphological change is the outcome of the selective binding of *β*-lactams to cellular surface protein components responsible for cell wall septum formation and separation of two divided organisms [66]. In addition, SEM images showed that different materials induced different levels of elongation on antibiotic-treated cells. To the best of our knowledge, this observation has never been described in the literature. The degree of cell elongation provides clues about cell susceptibility to the antibiotic treatment since ampicillin was more effective on biofilms formed on GLA, which was the surface material with the most elongated* E. coli* cells. Although SEM observations alone do not allow a predictive analysis on the antibiotic effectiveness, they are a remarkable support to understand the complex ecosystem which is the bacterial biofilm.

## 5. Conclusions

Through the high resolution imaging of colonized surfaces, it is possible to conclude that SEM is a valuable tool to study in detail the effect of antimicrobial agents on the cell morphology of bacterial populations in different biofilms. The proposed approach may be particularly useful in the case of biomedical devices containing mixed surfaces, where imaging the seals or transition zones can provide valuable hints about biofilm growth and antimicrobial susceptibility. Understanding this impact may provide clues for the modification of surface properties of biomedical materials as a strategy to increase the efficacy of antimicrobial therapy.

## Supplementary Material

The extent of cell removal from GLA and SIL coupons as a result of vortexing was 95% and 94%, repectively.
