# Supplementary material for: SEM Analysis of Surface Impact on Biofilm Antibiotic Treatment
Source: Scanning. 2017 Jan 11;2017:2960194. doi: 10.1155/2017/2960194 (PMC5662067; doi:10.1155/2017/2960194)
Supplement: Supplementary file 1 — The extent of cell removal from GLA and SIL coupons as a result of vortexing was 95% and 94%, repectively. [file 2960194.f1.docx]

**Supplementary Material**

Figure S1. Extent of total cell removal from GLA and SIL coupons determined by direct staining of 24 h-old biofilms with DAPI before (■) and after vortexing (□) for quantification of remaining cells. The means ± SDs for three sample surfaces are illustrated.
